# 

**Published:** 1906-03

**Authors:** 


### BOOKS RECEIVED.

Squibbs Materia Medica. Part I. A complete alphabetical list of all the Squibb products, embracing the articles in the U. S. Pharmacopeia (8th Revision) and the National Formulary, together with the non-official chemicals, pharmaceuticals, and newer remedies in general use. Part II. Squibb's Medicinal Tablets, etc. A reliable and comprehensive handbook for the Physician and the Pharmacist. 1906. edition. Brooklyn: E. R. Squibb & Sons.

State Commission in Lunacy for the State of New York. Sixteenth annual report, October 1, 1903 to September 30, 1904. Transmitted to the Legislature February 8, 1905. From Hon. Henry W. Hill, Senator 47th District. Albany: Brandow Printing Company. 1905.

Transactions of the Medical Association of the State of Alabama. (The State Board of Health). Organised 1847. Meeting of 1905 held at Montgomery, April 19-22, 1905. Montgomery: Brown Printing Company. 1905.

Food and Principles of Diatetics. By Robert Hutchinson, M.D., Edinburg, F.R.C.P.; Assistant Physician to the London Hospital, and to the Hospital for Sick Children, Great Ormond Street, etc. With plates and diagrams. Revised second edition. Octavo, 582 pages. New York: William Wood and Company. 1906. (Price: cloth, $3.00 net).

The World's Anatomists.' Concise Biographies of Anatomic Masters from 300 B. C. to the present time, whose names have adorned the literature of the medical profession. By G. W. H. Kemper, M.D., Professor of the History of Medicine in the Medical College of Indiana at Indianapolis. Revised and enlarged from the original serial publication in the Medical Book News. With eleven illustrations. Philadelphia: P. Blakiston's Son & Company. 1905.

Transactions of the American Gynecological Society. Volume 30, for the year 1905. J. Riddle Goffe, M.D., secretary. Philadelphia: William J. Dornan, printer. 1905.

A Treatise on Surgery. In two volumes. By George Ryerson Fowler, M.D., Examiner in Surgery, Board of Medical Examiners of the Regents of the University of the State of New York; Emeritus Professor of Surgery in the New York Polyclinic, etc. Two imperial octavos of 725 pages each, with 888 text illustrations and 4 colored plates, all original. Volume I. Philadelphia and London: W. B. Saunders Company. 1906. (Per set: Cloth, $15.00 net; half morocco, $17.00 net).

The Operating Room and the Patient. By Russell S. Fowler, M.D., Surgeon to the German Hospital, Brooklyn, N. Y. Octavo of 172 pages fully illustrated. Philadelphia and London: W. B. Saunders Company. 1906. (Cloth, $2.00 net).

A Textbook of Materia Medica, Theraputics, and Pharmacology. By George F. Butler, Ph.G., M.D., Associate Professor of Therapeutics in the College of Physicians and Surgeons, Chicago. Fifth Edition, thoroughly revised by Smith Ely Jelliffe, M.D., Ph.D., Professor of Pharmacognosy and Instructor in Materia Medica and Theraputics in Columbia University (College of Physicians and Surgeons), New York. Octavo of 694 pages, illustrated. Philadelphia and London: W. B. Saunders Company. 1906. (Cloth, $4.00 net; Half Morocco, $5.00 net).

A Textbook on the Practice of Gynecology. For Practitioners and Students. By W. Easterly Ashton, M.D., LL.D., Fellow of the American Gynecologic Society: Professor of Gynecology in the Medico-Chirurgical College of Philadelphia. Second Edition, revised. Octavo of 1079 pages, with 1046 original line drawings. Philadelphia and London: W. B. Saunders Company. 1906. (Cloth, $6.50 net; Half Morocco, $7.50 net).

Diseases of the Eye. A Handbook of Opthalmic Practice. By G. E. DeSchweinitz, M.D., Professor of Opthalmology in the University of Pennsylvania. Fifth edition, revised and enlarged. Octavo of 894 pages, 313 text-cuts and 6 chromo-lithographic plates. Philadelphia and London: W. B. Saunders Company. 1906. (Cloth, $5.00 net; Half Morocco, $6.00 net).

Reference Handbook of the Diseases of Children. For Students and Practitioners. By Professor Ferdinand Fruhwald, of Vienna. Edited, with additions, by Thompson S. Westcott, M.D., Associate Professor of Diseases of Children in the University of Pennsylvania, Octavo volume of 553 pages, with 176 illustrations. Philadelphia and London: W. B. Saunders Company. 1906. Cloth, $4.50 net; Half Morocco, $5.50 net).

Nursing in the Acute Infectious Fevers. By George P. Paul, M.D., Assistant Visiting Physician and Adjunct Radiographer to the Samaritan Hospital, Troy, N. Y. 12mo. of 200 pages, illustrated. Philadelphia and London: W. B. Saunders Company 1906. (Cloth, $1.00 net).

Essentials of Genito-Urinary and Veneral Diseases. By Starling S. Wilcox, M.D., Profesor of Genito-Urinary Diseases and Syphilology, Starling Medical College, Columbus, Ohio. 12mo. of 313 pages, illustrated. Philadelphia and London: W. B. Saunders Company. 1906. (Cloth, $1.00 net).

